# What treatment outcomes matter most? A Q-study of outcome priority profiles among youth with lived experience of depression

**DOI:** 10.1007/s00787-021-01839-x

**Published:** 2021-07-17

**Authors:** Karolin Rose Krause, Julian Edbrooke-Childs, Holly Alice Bear, Ana Calderón, Miranda Wolpert

**Affiliations:** 1 Evidence Based Practice Unit (EBPU), Anna Freud National Centre for Children and Families, 4-8 Rodney Street, London, N1 9JH UK; 2 Research Department of Clinical, Research Department of Clinical, University College London, Gower Street, London, WC1E 6BT UK; 3 Cundill Centre for Child and Youth Depression, Centre for Addiction and Mental Health (CAMH), 80 Workman Way, Toronto, ON M6J 1H4 Canada; 4 Department of Psychiatry, University of Oxford, Warneford Hospital, Warneford Lane, Oxford, M6J 1H4 UK; 5 School of Psychology, Universidad Gabriela Mistral, Avda. Ricardo Lyon 1177, Providencia, Santiago, Chile; 6 Wellcome Trust, 215 Euston Rd, Bloomsbury, London, NW1 2BE UK

**Keywords:** Adolescents, Depression, Q-methodology, Treatment outcomes, Outcome priorities

## Abstract

Interest in youth perspectives on what constitutes an important outcome in the treatment of depression has been growing, but limited attention has been given to heterogeneity in outcome priorities, and minority viewpoints. These are important to consider for person-centred outcome tracking in clinical practice, or when conducting clinical trials targeting specific populations. This study used Q-methodology to identify outcome priority profiles among youth with lived experience of service use for depression. A purposive sample of 28 youth (aged 16–21 years) rank-ordered 35 outcome statements by importance and completed brief semi-structured interviews eliciting their sorting rationales. By-person principal component analysis was used to identify outcome priority profiles based on all Q-sort configurations. Priority profiles were described and interpreted with reference to the qualitative interview data. Four distinct outcome priority profiles were identified: “Relieving distress and experiencing a happier emotional state”; “Learning to cope with cyclical distressing emotional states”; “Understanding and processing distressing emotional states”; and “Reduced interference of ongoing distressing emotional states with daily life”. All four profiles prioritised improvements in mood and the ability to feel pleasure but differed in the level of importance assigned to learning coping skills, processing experiences, and the reduced interference of depression with life and identity. As part of a person-centered approach to care delivery, care providers should routinely engage young people in conversation and shared decision-making about the types of change they would like to prioritise and track during treatment, beyond a common core of consensus outcomes.

**Supplementary Information:**

The online version contains supplementary material available at 10.1007/s00787-021-01839-x.

## Introduction

Depression is a common and serious mental health problem in adolescence, with lifetime prevalence rates of 11–16% in the United States and Europe [1–3]. Unless treated effectively, adolescent depression can have negative impacts on mental and physical well-being, educational attainment, employment, and income across the life course [4–10] There is a need to enhance the efficacy of available treatment options, as well as their effectiveness in clinical practice. Within a framework of person-centred care, treatment should enable change in outcomes that matter to service users and their families [11, 12]. These outcomes should be tracked in clinical trials and routine practice to ensure that treatment efficacy and effectiveness are appraised in relation to service user priorities. Yet, outcome priorities of young people have historically received limited attention. Measurement approaches have generally been determined with minimal youth input, and have tended to focus on symptomatic change [13–15].

This is beginning to change. Several recent consultations and qualitative studies have begun to examine young people’s perceptions of “good outcomes” in the context of mental health treatment. In the United Kingdom (UK), two studies examined treatment goals defined by youth with diverse mental health difficulties in the context of specialist mental health care [16] and school-based counselling [17]. Frequent goal themes related to improvements in symptom severity, coping skills, daily functioning, personal growth (e.g., increased self-confidence and self-acceptance) and improved interpersonal relationships. In Norway, semi-structured interviews and focus groups with youth treated at specialist mental health clinics identified five “good outcome” themes that related to understanding and coping with emotions; developing an identity that is independent from social pressures and mental health conditions; reaching out and relating to others; being able to accomplish goals and embrace new challenges; and developing the hope, optimism and agency needed to cope in the long term [18].

Two qualitative studies have examined outcome perceptions specifically for youth experiencing depression. Krause and colleagues [19] conducted a qualitative content analysis of young people’s change narratives following their participation in a psychotherapy  trial [20, 21]. The most frequently discussed outcome themes related to reductions in core depressive symptoms (i.e., mood and affect), and coping skills and resilience, each discussed by two thirds of youth. Other prominent themes related to improvements in family functioning and relationships, feeling seen and seeing differently, and improved social functioning. A qualitative study from Chile examined outcome perceptions among six depressed adolescents [22], and identified themes such as improved well-being, greater calm, and enhanced motivation and assertiveness in addition to symptomatic change, learning to cope, and improved family interactions.

While these existing qualitative studies have showcased a rich variety of outcomes that are valued by youth, less is known about how outcome priorities differ between youth, although there is some evidence of heterogeneity in outcome perceptions. A narrative analysis of young people’s experiences with school-based counselling suggests considerable heterogeneity: Youth demonstrating a “transformative” narrative described profound changes to the self; youth with a “supportive” narrative described counselling as mainly holding them in place; youth showcasing a “pragmatic” narrative suggested counselling had helped with solving specific problems; and youth with a “disappointed” narrative were unable to describe any positive outcomes enabled by counselling [23]. Recent work to develop a conceptual framework of recovery narratives for adult mental health has also identified a high degree of heterogeneity with regards to the emotional tone of recovery narratives, individuals’ perceived relationships with and trajectories of recovery, and any perceived turning points [24, 25].

Similarly, to the best of our knowledge, no existing qualitative study has yet specifically examined which treatment outcomes youth with depression consider *most* important. The prioritisation of outcomes is of growing interest due to the recent emergence of Core Outcome Set initiatives for youth depression. Core Outcome Sets recommend a minimum set of outcomes to be measured across all research studies or practice settings for a given condition, with the aim to promote the harmonised measurement of outcomes that matter to key stakeholders [26, 27]. One recently developed Core Outcome Set recommends tracking symptom severity, functioning, and suicidal thoughts and behaviour, as a minimum, for depressed youth treated in clinical practice settings [28]. A second set focussing on youth depression clinical trials is currently under development [29]. Both initiatives have engaged young people in the selection of core outcomes. However, by their nature, consensus-building initiatives focus on identifying common ground, and may offer limited scope to incorporate minority viewpoints. Yet, within a person-centered care framework, individual outcome priorities are important to consider alongside consensus outcomes. An improved understanding of different priority profiles could inform conversations and shared decision-making about outcome measurement with youth in clinical practice, as well as the selection of outcomes for clinical trials.

### The present study

This study aimed to build upon existing qualitative research and consultations about young people’s outcome perceptions and priorities, by using Q-methodology—a method that is tailored to the systematic study of subjective viewpoints [30, 31]. Q-methodological studies typically invite participants to sort an item set according to a pre-defined ranking scheme (e.g., by importance) [32]. Inverted factor analysis is then used to identify distinct viewpoints based on the participants’ sorting patterns. The viewpoints are then described and interpreted qualitatively. The technique moves beyond eliciting a majority view by identifying a range of perspectives, including minority experiences [33, 34]. Q-methodology has been deployed in adolescent health contexts to examine preferences for hospital care among youth with chronic physical health conditions [34]; antidepressant side effects [35]; reasons for medication non-adherence following renal transplants [36, 37]; and attitudes towards health-related lifestyles among non-clinical youth [38]. To our knowledge, no Q-study has yet examined outcome priorities in relation to adolescent depression. This study was part of a wider Q-methodological research project into outcome priorities for adolescent depression, which included parallel investigations with mental health practitioners in the United Kingdom (UK) and in Chile.

## Method

### Participants

Q-methodological studies aim to identify and describe viewpoints in depth, rather than assert their prevalence in a reference population [39]. As subtle patterns of meaning become difficult to analyse with increasing data volumes [40, 41], Q-studies typically involve between 20 and 50 purposively sampled participants. In inverting sample size guidelines for traditional factor analysis, it is recommended that the number of participants should not exceed the number of items to sort [42, 43]. As the present Q-study employed a 35-item Q-set (see below), we aimed for a maximum participant sample of 30 young people, but used a principle of saturation: once a minimum of 25 participants had been recruited, additional interviews were conducted until no substantially new viewpoints were articulated [44].

Given limited evidence around the demographic and clinical characteristics influencing youth outcome priorities, sampling was not hypothesis-led. Instead, we aimed to represent varying depression histories and socio-demographic profiles. Adolescents were eligible if they were aged 12 to 21 years, and self-identified as having lived experience of accessing mental health support for depression. Co-occurring presenting problems (including neurodevelopmental disorders) were not an exclusion criterion, so long as young people were able to complete the research tasks in a self-directed manner. However, youth known to be experiencing acute suicidal ideation or psychosis at the time of recruitment were excluded for safeguarding reasons. Using convenience sampling [45], we advertised recruitment calls through the networks of youth mental health charities in England; by soliciting youth peer support groups across England; through the University College London Psychology Subject Pool; and through social media.

The Q-sort was completed by a volunteer sample of 28 adolescents who were aged between 16 and 21 years. As intended, the sample was diverse with regards to socio-demographic profiles, experiences of service use, and the type of mental health difficulties they had experienced, according to self-report. An overview of these characteristics is provided in Table 1. Eighteen youth (64%) identified as female, nine identified as male (32%) and one identified as non-binary. The mean age was 18.7 years. All young people identified as having lived experience of accessing some form of mental health support for depression. Around half reported that they were participating in ongoing mental health treatment (43%) while the remainder reported a history of past treatment. Half of the sample (54%) stated that they had engaged in more than one cycle of treatment. According to self-report, one in four youth had been admitted to emergency care in relation with their depression, and three (11%) had accessed inpatient care. The majority (86%) stated that they had participated in individual therapy or counselling, and more than half reported that they had been prescribed medication as part of a prior or ongoing treatment (57%).

**Table 1:** Demographic characteristics

Variable	Overall
*N*	28
Gender	
Female	18 (64%)
Male	9 (32%)
Non-binary	1 (4%)
Mean age (SD)	18.7 (1.8)
Treatment history (based on self-report)	
Treatment ongoing (vs. ended)	12 (43%)
Repeated cycles of treatment (vs single cycle)	15 (54%)
History of admission to emergency care	7 (25%)
History of inpatient care	3 (11%)
Average number of additional difficulties as per self-report (SD)	4.1 (2.4)
History of additional mental health difficulties (based on self-report)	
Anxiety or phobia	23 (82%)
Sleeping problems	18 (64%)
Self-harm	17 (61%)
Eating problems	16 (57%)
Neurodevelopmental disorder (e.g., ADHD, ASD)	8 (29%)
Learning difficulties (e.g., dyslexia or dyspraxia)	6 (21%)
Anger and violent behaviour	6 (21%)
Obsessions or compulsions	5 (18%)
Substance use	5 (18%)
Psychosis	5 (18%)
Trauma	4 (14%)
Types of treatment received (based on self-report)	
Individual psychotherapy or counselling	24 (86%)
Medication	16 (57%)
Family therapy	13 (46%)
Group therapy	8 (29%)

*ADHD* attention deficit hyperactivity disorder, *ASD* autism spectrum disorder

To gain an indication of the complexity of their lived experience, youth were asked to indicate whether there were other mental health difficulties, beyond depression, that they had discussed with a mental health professional. Most commonly, youth reported that they had discussed difficulties related to anxiety (82%), disrupted sleep (64%), self-harm (61%), and disordered eating (57%). Less frequent difficulties included issues with anger and aggression, obsessions and compulsions, substance use, and psychosis (see Table 1). In addition, 29% of youth reported difficulties related to a neurodevelopmental disorder like attention deficit hyperactivity disorder (ADHD) or autism spectrum disorder (ASD), and 21% reported learning difficulties (e.g., dyslexia or dyspraxia). Young people did not complete a structured psychometric assessment of current depressive or co-occurring symptoms, since having any self-reported lived experience of treatment for depression rather than current symptom status was the principal inclusion criterion.

### Procedure

To create the item Q-set, we first compiled a candidate pool of outcomes through two interdisciplinary workshops involving young people; an interdisciplinary discussion group with researchers from the fields of psychology, psychometrics, the social sciences, and philosophy; a systematic literature review of treatment outcome studies for adolescent depression [15]; and qualitative content analysis of 102 semi-structured interviews with adolescents, their parents, and therapists conducted as part of the IMPACT psychotherapy trial for depression [19]. This process identified 73 possible outcomes, across the eight domains of *symptoms, coping and self-management*, *functioning*, *personal growth*, *relationships*, *therapeutic space*, *youth well-being*, and *parental support and well-being.* Outcome descriptions were refined and harmonised, and redundant items removed or collapsed [42]. Using Fisherian balanced block design, we selected four to five outcomes from each domain [46, 47] to obtain a structured 35-item Q-set [48]. While it is recommended that Q-sets include between 40 and 80 items, smaller sets have been favoured for use with children and young people [34, 36, 38, 49]. An early draft of the Q-set was piloted with two youth advisors, and a close-to-final version was reviewed for the clarity of the language and concepts by an adolescent volunteer. After final adjustments, each outcome description was printed on a separate numbered card for sorting.

**Fig. 1:**
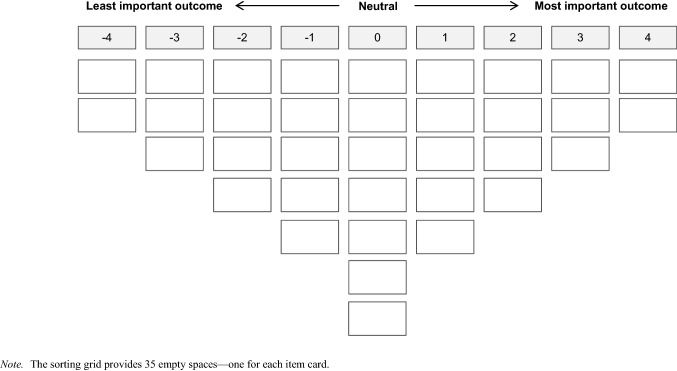
Sorting grid used by participants

We invited participants to rank order the 35 outcome descriptions according to a quasi-normal distribution, using a sorting grid with a 9-point scale of importance (from + 4 *most important,* to − 4 *least important,* see Fig. 1). Following completion, participants answered open-ended questions about the rationale for their outcome prioritisation, and could indicate whether they felt that any important outcomes were missing [50]. These brief post-sort interviews were recorded and transcribed verbatim. Participants also completed a brief demographic questionnaire capturing their self-reported history of depression and co-occurring mental health difficulties. Half of the participant sample (*n* = 14) completed the card sorting at individual appointments; the remainder completed the task individually at a peer support group meeting. All post-sort interviews were conducted in a confidential one-on-one setting. Adolescents were remunerated for their time with £10 gift vouchers and reimbursed for travel expenses.

### Statistical analysis

Clusters of adolescents whose Q-sorts were highly correlated were identified via inverted (or “by-person”) principal component analysis (PCA), using the dedicated software PQMethod [51]. We identified the most suitable component solution by considering the scree plot of Eigenvalues, the shared variance explained, the number of Q-sorts loading significantly on only one principal component, and the correlation between component scores. To improve model fit, the unrotated correlation matrix was first subjected to Varimax rotation [52] to identify a solution that would mathematically maximise the variance explained [42], and small adjustments via hand rotation were then applied to increase the number of participant Q-sorts loading significantly on exactly one of the components [42].

For each extracted principal component (i.e., outcome priority profile), an ideal–typical Q-sort was generated by averaging the outcome rankings across all participant Q-sorts associated within the given component, using the correlation coefficients as weights [38]. The ideal–typical Q-sort illustrates how an archetypical profile representative would have rank-ordered the 35 outcomes [42]. Outcome profiles were interpreted with reference to this ideal–typical Q-sort, and by drawing on the qualitative data collected through post-sort interviews with the participants, which were analysed using qualitative content analysis [53].

### Ethical considerations

This study was approved by the ethics review committee of University College London in March 2018 (UCL REC REF: 10567/002) and performed in accordance with the ethical standards of the committee and with the 1964 Helsinki declaration and its later amendments or comparable ethical standards. All participants were above the age of 16 and provided informed written consent. All interview data were anonymized, and names replaced with pseudonyms. Hereafter, participants are identified by their pseudonyms and without indication of their age to provide the highest-possible level of confidentiality.

## Results

By-person PCA identified four outcome priority profiles that explained 48.7% of the common variance in youth Q-sorts. Inter-component correlations ranged from 0.05 to 0.33, without reaching statistical significance at *p* < 0.01, suggesting that distinct preference profiles had been extracted. Two Q-sorts did not load significantly on any principal component; and one was confounded (i.e., had significant loadings on two components). These Q-sorts were not considered for further analysis [42]. The rotated component matrix and loadings are shown in Table 2. Hereafter, we describe each of the four outcome priority profiles, as well as any consensus statements that received similar rankings across profiles. We refer to the ideal–typical Q-sorts (see Table 3) by providing statement numbers and ranks in parentheses (e.g., #3, + 3), and draw on the post-sort interviews for interpretation. Figure 2 provides an illustration of the outcomes considered most and least important by each profile. An overview of socio-demographic characteristics and treatment history by profile is provided in the Supplement (Table S1).

**Table 2:** Component loadings for each outcome priority profile

Outcome priority profile	Participant Q-sort^a^	Component loadings for each profile			
		A	B	C	D
Profile A: relieving distress and experiencing a happier emotional state	Becca	**0.80**	0.35	0.14	0.13
	Dylan	**0.79**	0.18	−0.09	0.23
	Ellie	**0.69**	0.09	0.18	−0.19
	Soraya	**0.62**	0.16	−0.04	−0.11
	Samuel	**0.61**	0.30	0.37	0.25
	Josh	**0.49**	0.44	0.33	0.07
Profile B: learning to cope with cyclical distressing emotional states	Melody	−0.28	**0.77**	0.14	−0.04
	Adam	0.09	**0.68**	−0.39	0.24
	Ameera	−0.05	**0.63**	0.30	0.13
	Jacob	0.37	**0.54**	0.10	−0.43
	Hannah	0.26	**0.52**	−0.24	−0.02
	Liam	−0.07	**0.51**	0.16	0.00
	Taylor	0.08	**0.47**	0.28	0.36
	Boris	0.26	**0.44**	0.32	0.16
Profile C: understanding and processing past and present distressing emotional states	Lauren	−0.01	0.06	**0.74**	0.05
	Chelsea	0.04	−0.12	**0.67**	0.35
	Imogen	0.03	−0.02	**0.61**	−0.20
	Liz	−0.23	0.15	**0.53**	−0.04
	Connor	−0.03	0.44	**0.51**	0.02
	Jade	0.39	0.28	**0.50**	−0.22
	Chloe	0.26	0.33	**0.49**	−0.18
	Amber	0.18	0.29	**0.44**	0.32
Profile D: reduced interference of ongoing distressing emotional states with daily life	Lewis	0.04	−0.05	−0.05	**0.66**
	Georgia	−0.27	0.19	0.00	**0.65**
	Meghan	0.25	−0.07	016	**0.51**
Not assigned	Karimah^b^	−0.09	0.55	0.54	−0.09
	Faizah^c^	0.29	−0.05	0.06	0.04
	Lien^c^	0.03	0.25	0.11	−0.25
Variance explained (%)		13%	15%	14%	8%
Composite reliability		0.96	0.97	0.97	0.92

^a^All names are pseudonyms. ^b ^This Q-sort had significant loadings on two principal components (i.e., was confounded). ^c^ This Q-sort did not load significantly on any of the four principal components

**Table 3:** List of Q-set items with composite principal component scores (i.e., ideal–typical item rank)

# Q-sort item		Item rank			
		Profile A	Profile B	Profile C	Profile D
Symptoms					
1	Being less angry and not losing my temper as much	−2	−4	−1	0
2	Feeling less down and depressed	**4**	2	2	**3**
3	Feeling happier and enjoying things more	**4**	**4**	1	**4**
4	Feeling more loved	**3**	1	−2	−2
5	Engaging less in behaviour that can be harmful	**3**	−4	**4**	0
Coping and self-management					
6	Being more active and engaged in things	0	−2	0	−2
7	Knowing ways to cope with my emotions	**3**	**3**	1	−4
8	Having a better understanding of my feelings and thoughts	0	**3**	0	−1
9	Being able to challenge negative thoughts and approach situations differently	2	**4**	1	−3
Functioning					
10	Being better able to get things done (e.g., concentrate, be organised)	1	0	−2	0
11	Being able to do the same things other adolescents do	−2	−2	−3	**4**
12	Working more effectively in school (e.g., being more motivated and focussed)	0	−1	−1	0
13	Attending school more regularly	−2	0	−3	−3
14	Being more sociable and better able to be around other people	2	−1	−3	1
Personal growth					
15	Feeling more confident	1	0	0	**3**
16	Being better able to stand up for my needs and opinions	−1	−3	0	−2
17	Being more independent and able to take responsibility for my life	−1	2	0	0
18	Being able to make sense of things that have happened in the past, or that are still happening	1	2	**3**	1
19	Having a better sense of who I am and how to be myself around others	0	1	−1	1
Relationships					
20	Feeling more able to talk about my feelings and thoughts	−1	**3**	−1	2
21	Getting on better with my family	0	0	2	−4
22	Getting on better with my friends or having made new friends	1	−3	−4	−1
23	Getting on better with my peers in school (e.g. not feeling bullied)	−2	−3	−4	−3
Therapeutic space					
24	Having a space where someone listens and cares about me	−1	−2	**3**	−2
25	Having a space where I can let out my feelings	−1	0	−1	2
26	Having a space where I can talk about anything without being judged	0	1	1	−1
27	Having a space to reflect and think about things differently	−3	0	0	−1
Well-being					
28	Having greater peace of mind (e.g. feeling calmer, more balanced)	2	1	2	−1
29	Feeling more optimistic and positive about life and the future	2	2	**4**	2
30	Feeling physically healthier	0	−2	−2	0
31	Being able to make plans for the future and have goals	1	0	2	2
Parental support and well-being					
32	My parents feeling happier and less stressed and worried	−3	−1	0	1
33	My parents having a better understanding of me and my difficulties	−3	1	**3**	0
34	My parents feeling more able to support me	−4	−1	1	1
35	My parents feeling less guilty	−4	−1	−2	**3**

**Fig. 2:**
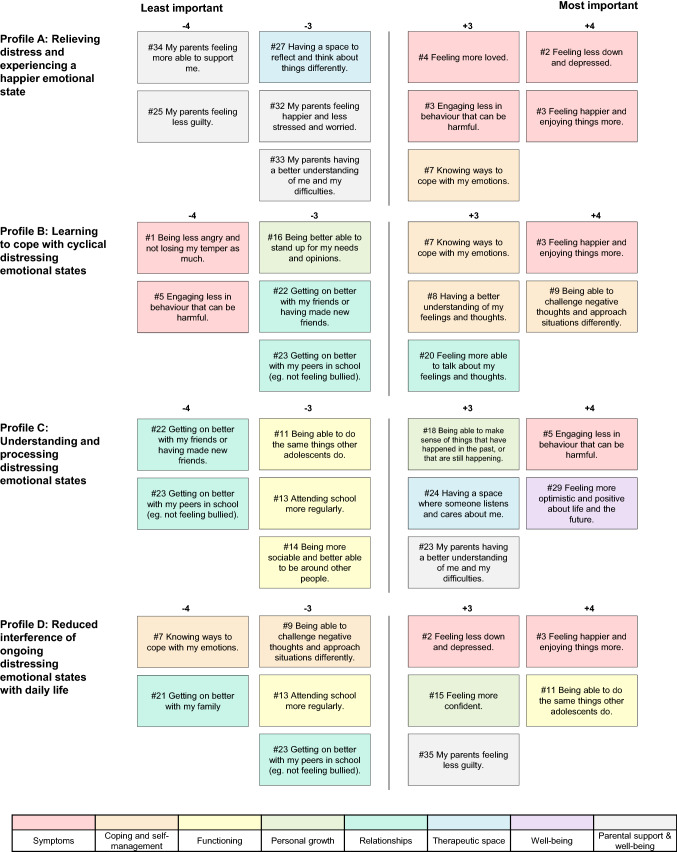
Outcome statements ranked as most and least important by each outcome priority profile

### Common outcome themes across youth profiles

There was consensus among all four outcome priority profiles on the importance of reducing core depressive symptoms such as low mood and the loss of pleasure and enjoyment. Feeling happier and enjoying things more (#3) was ranked as a *most* important outcome (rank + 4) by three out of four profiles; and feeling less down and depressed (#2) was considered very or somewhat important (ranks + 2 to + 4) by all. In addition, all four profiles assigned a high level of importance to feeling more optimistic and positive about life and the future (#29, + 2 to + 4). Most consistently ranked as unimportant were improved relationships with peers in school (#23, − 2 to − 4), with many youth explaining that they did not struggle with peer relationships or bullying.

#### Profile A: relieving distress and experiencing a happier emotional state

Profile A represents six youth and explains 13% of the common variance. A primary desire for this profile was to overcome the distressing emotional states typically associated with depression. Young people prioritised emotional changes such as feeling less down and depressed, feeling happier and enjoying things more (#2 and #3, + 4), and feeling more loved (#4, + 3). One young person described: “I’d been feeling it for so long it was like something that I just wanted to get rid of. And especially because like ‘feeling less down and depressed,’ for a depressed person that seems like … heaven” (Becca).

Other priority outcomes also related to reaching a happier emotional state and included learning strategies to cope with emotions (#7, + 3), reducing potentially harmful behaviour (#5, + 3), having greater peace of mind (#28, + 2) and feeling more optimistic about life and the future (#29, + 2). Compared with other outcome profiles, these youth gave higher importance to becoming more sociable (#14, + 2) and getting on better with friends (#22, + 1), as they tended to describe their depression as a barrier to connecting with others. In turn, youth deprioritized outcomes related to parental support and parental well-being (#32–35, − 3 to − 4), and explained that they preferred to manage their difficulties independently or felt that their parents had a limited ability to understand and support them.

Youth in this profile had a mean age of 18.2 years (SD = 1.6); half were female. With one exception, these youth were not currently receiving treatment, but half reported that they had engaged in multiple treatment cycles. One young person had been admitted to emergency care in relation with their depression, but none had been in inpatient care. Youth reported lived experience with four additional mental health difficulties, on average, including anxiety (*n* = 6), sleep problems (*n* = 4), self-harm (*n* = 4), and disordered eating (*n* = 4). Difficulties mentioned less frequently included a neurodevelopmental disorder, problems with anger or aggression, obsessions or compulsions, substance use, and psychosis. All adolescents reported experience of individual psychotherapy, four reported experiences of taking antidepressant medication, and two each reported experience with family or group therapy.

#### Profile B: learning to cope with cyclical distressing emotional states

Profile B represents eight youth and explains 15% of the common variance. Although improved mood was a priority outcome (#3, + 4), these youth did not believe that treatment would provide an ultimate cure and enable them to reach a happier emotional state once and for all. Instead, they described their depression symptoms as cyclical and prioritised a range of outcomes related to gaining the coping skills and agency required to manage their symptoms independently in the long-term. These outcomes included learning to challenge negative thoughts and approach situations differently (# 9, + 4), learning strategies to cope with emotions (#7, + 3), having a better understanding of feelings and thoughts (#8, + 3), and feeling more able to talk about feelings and thoughts (#20, + 3). By acquiring these skills, youth hoped that they would be able to prevent or manage future depressive episodes:

*Like when the first wave of sadness hits, normally you don’t have the strategies, you will just […] be snowballing, but […] it’s important to find ways to kind of break that momentum and stop that snowball before it just gets worse.* (Jacob)

Youth in this profile were reluctant to become reliant on professional help and valued outcomes related to being independent and able to take responsibility for their own lives (#17, + 2):

*When I first went to CAMHS [child and adolescent mental health services], it was a case of I just wanted to not feel this way anymore. But when I kept going back to CAMHS, then I thought, this isn’t sustainable, I need to be able to function without CAMHS so the more times I cycled through getting help, the more important sort of resilience or being able to help myself became.* (Hannah)

The outcomes ranked as least important related to aspects of life that youth in this profile felt they did not struggle with, such as managing anger (#1, − 4), risky or harmful behaviour (#5, − 4), family and peer relationships (#22 and 23, − 3), or a lack of assertiveness (#16, − 3).

Youth in this profile had a mean age of 19.3 years (SD = 2.2); three out of seven were female. Half reported being in treatment at the time of the research; five out of eight reported having participated in several cycles of treatment. None of the youth reported a history of accessing emergency or inpatient care in relation with their depression. On average, youth described three additional mental health difficulties, with the most common ones being anxiety (*n* = 5), eating or sleep problems (*n* = 4), and self-harm (*n* = 3). Less frequently mentioned co-occurring problems included neurodevelopmental disorders, obsessions or compulsions, substance use and psychosis. None of the youth reported a history of trauma, or a struggle with anger or aggression. Youth reported experience of individual psychotherapy (*n* = 7), family therapy (*n* = 4), antidepressant medication (*n* = 3), and group therapy (*n* = 2).

#### Profile C: understanding and processing distressing emotional states

Profile C represents eight youth and explains 14% of the common variance. This profile focussed on finding safe outlets for emotions, making sense of past and present experiences, and gaining a more positive outlook into the future. A considerable share of young people representing this viewpoint had sought mental health support not just for depression, but also in relation to overlapping needs, including learning difficulties (*n* = 4), ASD (*n* = 3), ADHD (*n* = 2) or trauma (*n* = 2). These youth tended to feel that growing up with these difficulties had set their experiences apart from those of peers or family members, and they often struggled to make sense of these experiences themselves:

With the Asperger’s, I don’t really understand emotions in general […] I can never tell if I’m sort of truly feeling something or if I’m just thinking I’m feeling that. (Jade)

Young people with ASD in particular described anxieties about the future and their prospects for accessing higher education or employment, which in turn would affect their mood. Youth frequently described self-harm as an outlet for overwhelming emotions that they could not articulate or manage otherwise. In this context, youth endorsed a mix of outcomes that revolved around calming some of the anxieties and confusion that stemmed from experiencing the world differently, and around trying to make sense of their emotional states in order to be able to move forward:

I kind of wanna get all my thoughts in order and there’s a lot of stuff that has happened in the past that I wanna deal with before I start dealing with stuff now. (Chelsea)

In line with this, the most highly ranked outcomes included finding safe outlets for their emotions and reducing self-harm (#5, + 4), being able to make sense of past and current experiences (18, + 3), feeling more optimistic about life and the future (#29, + 4), and having greater peace of mind (#28, + 3). Youth longed to feel better understood by their parents (#33, + 3), to improve their family relationships (#22, + 2), and to have a space where somebody listened and cared about them (#24, + 3). In contrast, outcomes relating to psychosocial functioning (#11, 13, 14; rank − 3) and peer relationships (#22 and 23, − 4) were assigned low importance, with several youth explaining that they did not strive to be “typical” adolescents and that they felt at ease with a select group of friends or with being by themselves.

Youth in this profile had a mean age of 18.8 years (SD = 1.8); seven were female. Half reported that they were still receiving treatment; half had engaged in several courses of treatment; three had visited emergency care in relation with their depression; and one had spent time in inpatient care. On average, youth reported five additional mental health difficulties, with anxiety being the most frequent (*n* = 7), followed by self-harm (*n* = 6), sleep problems (*n* = 5) and neurodevelopmental disorders (*n* = 4), learning difficulties (*n* = 4), and obsessions or compulsions (*n* = 3). Anger and aggression, substance use, psychosis, and trauma were less frequently mentioned. Youth reported experience of individual psychotherapy (*n* = 6), antidepressant medication (*n* = 6), family therapy (*n* = 4) and group therapy (*n* = 2).

#### Profile D: reduced interference of ongoing distressing emotional states with daily life

Profile D represents three youth and explains 8% of the common variance. This profile revealed an experience marked by a constant struggle with a complex set of mental health difficulties, and a desire to recover a life and identity that would not be defined by this struggle.

Youth in this small group described considerable impairment that often had been present for years. This included, for example, having to interrupt school, being unable to go out with friends, or to use public transport. As described by Georgia: “*It’s affected everything, like literally everything*.” To this group, feeling happier and enjoying things more (#3, + 4), and being able to engage in age-typical activities (#11, rank + 4) were the two most important outcomes. Feeling less down and depressed was also highly ranked (# 2, + 2). In addition, these youth prioritised recovering a sense of confidence and hope (#15, + 3; #29 and 31, + 2) to envisage an identity and future beyond their struggle with their mental health:

“Who I am can feel quite dependent on my mood at that moment, and if I’m feeling very low then I’m like […] nothing’s ever gonna be worth it…” (Meghan).

These youth also worried about the impact that this struggle had had on their families and were the only profile to prioritise a reduction in parental guilt as an important outcome (#35, + 3). Contrary to other profiles, these youth did not prioritise improvements in coping and self-management (#6–9, − 1 to − 4), expressing scepticism that such strategies could be deployed at will, especially when emotions became overwhelming.

The mean age of youth in this profile was 19.3 years (SD = 1.5); two were female. Two reported that they were receiving ongoing treatment; two had engaged in several episodes of treatment; and two reported that they had been admitted to both emergency care and inpatient care in relation with their depression. Youth in this profile reported the highest burden from additional mental health difficulties (in seven areas on average) out of all profiles. All three reported anxiety, self-harm, and problems with eating and sleep. Additional difficulties mentioned by one or two youth included a history of psychosis, trauma, and issues with anger. All youth reported that they had participated in individual psychotherapy, and two each had been treated with medication, family therapy, or group therapy.

#### Additional outcomes

Youth suggested a number of additional outcomes that they felt were missing from the Q-set. Overcoming a sense of boredom or numbness, and finding an interest in something (e.g., a hobby or project for the future) was mentioned by two young people. Other additional outcomes were mentioned by one young person each and included improved sleep, reduced feelings of loneliness, overcoming a sense of personal guilt for the impact that one’s depression may have had on friends and family, developing the ability to trust and confide in others outside of therapy, a general sense of well-being, being able to discontinue medication, and improved productivity at work.

## Discussion

This Q-study demonstrates plurality in young people’s views about the outcomes that are most important when receiving treatment for depression. Four distinct outcome priority profiles were identified: (A) Relieving distress and experiencing a happier emotional state; (B) Learning to cope with cyclical distressing emotional states; (C) Understanding and processing distressing emotional states; and (D) Reduced interference of ongoing distressing emotional states with daily life. The four profiles aligned in assigning high priority to improvements in mood and the ability to feel pleasure, which is in line with prior research emphasizing the reduction of emotional distress as a priority outcome for youth with depression [19]. However, profiles differed with regards to other outcome priorities.

Profile A expressed a desire to overcome depressive symptoms once and for all, while Profile B conveyed a cyclical understanding of depression and prioritised the acquisition of coping skills. The importance of learning to cope has been a common theme in previous qualitative studies with youth [17–19, 22, 54, 55], including a recent study of youth’s cognitive illness representations that identified a similar linkage between perceptions of depression as a cyclical long-term condition and a focus on self-management [56]. Youth in Profile C were disproportionately affected by neurodevelopmental and learning difficulties and struggled with ongoing emotional distress. Making sense of their experiences, finding safe outlets for their emotions, and feeling understood were priority outcomes for this group. They did not prioritise outcomes related to functional impairment or convey a desire to be more similar to their peers, which resonates with suggestions that being different does not necessarily equate to being or feeling impaired (although it may cause distress) [57]. In contrast, Profile D represents a small group of youth with complex ongoing mental health difficulties for whom a reduction in functional impairment was a principal priority.

These four profiles convey not only different outcome priorities, but also different positions with regards to the anticipated room of opportunities for improvement. Both outcome priorities and room for improvement appear to vary with the types and complexity of mental health difficulties youth have experienced, as well as with the resilience resources they have available [58]. They also appear to vary with regards to young people’s beliefs about the timelines of their depression (e.g., acute, chronic, cyclical), their perceived locus of control over symptoms, and the perceived effectiveness of treatment in durably alleviating symptoms. These factors have previously been described to shape an individual’s cognitive representation of a health condition, as well as their approach to managing it, including in relation to youth depression [56, 59, 60].

 In the present study, young people who prioritised improved coping and self-management skills had less complex mental health histories than youth in other profiles, reporting fewer co-occurring difficulties, and no history of admission to emergency or inpatient care. Contrary to youth in profiles A and C, they tended to feel supported by family and friends; and contrary to profiles C and D, they did not feel held back by a lack of self-confidence. More than half of these youth had successfully navigated their way towards professional support on multiple occasions. This group thus appeared relatively well resourced to manage depressive symptoms—perceived as cyclical—without ongoing professional support. In contrast, coping and self-management outcomes were deprioritized by youth in Profile D who did not convey the same optimism as Profile A with regards to becoming symptom-free; nor did they share the self-efficacy of youth in Profile B. For these youth, complex emotional distress was omnipresent, interfered considerably with their ability to cope and carry out daily activities, and often felt beyond their control.

If outcome priority profiles reflect different mental health profiles and trajectories, they may have at least some stability over time, as opposed to being purely temporary expressions of current symptom levels, although new experiences may well inform outcome priorities on an ongoing basis. Developmental and life stage considerations, cultural influences, and socio-economic factors may also shape young people’s judgements of what outcome is desirable and achievable. Goal setting research shows that youth aspirations are shaped by age, gender, family characteristics, and ethnicity; the socio-political environment; as well as cultural and gender norms [61, 62]. Additional research is needed to explore how outcome priorities evolve over time, and to what extent they are influenced by current symptom levels, cognitive representations of depression e.g., [56], and socio-cultural factors.

It is of note that none of the four profiles consistently prioritised outcomes related to personal growth, for example in terms of improved confidence, assertiveness, and independence, which constitute central themes within the recovery paradigm [63]. The recovery literature originates in adult mental health, and it is possible that the youth in this sample did not actively think of these outcomes as immediately important or within their reach. Nonetheless, personal growth outcomes were a key theme in at least one consultation with youth of a similar age who had experienced mixed mental health difficulties [18]. In the present study, improving self-confidence was ranked highly only by youth in profile D, while greater independence was ranked highly only by youth in profile B. This showcases a degree of heterogeneity in priorities that can be masked by inquiries that focus on general themes or narratives.

### Implications

Our findings suggest that improvements in core depressive symptoms such as improved mood or ability to feel pleasure are highly valued by most young people with experience of depression. This supports the inclusion of symptom measures in consensus-based Core Outcome Sets for youth depression [28]. At the same time, our findings suggest there is no one-size-fits all outcome prioritisation, as youth diverge on the additional outcomes they value the most. For the purpose of person-centered assessment, services should aim to identify personal priority outcomes through conversation and shared decision-making with young people, and consider tracking these personal priority outcomes alongside symptom change [64, 65]. This can be done via validated standardised scales assessing coping skills or global functioning, or via personalised outcome measures such as the Goal-Based Outcome Measure [66] or the Youth Top Problems [67]. The latter provide opportunities to track idiographic outcomes [68–70], as well as outcomes that are not typically covered by standardised scales, such as being able to talk about feelings and thoughts, understanding emotions, taking greater responsibility for oneself, feeling more confident, or having a clearer understanding of one’s identity and past [71].

Our findings suggest that young people’s outcome priorities appear to be influenced by the type and complexity of mental health difficulties that youth have experienced, the resources they have available, and cognitive representations of the condition. Future research is needed to better understand the relationships between these factors and young people’s outcome priorities, as well as how such knowledge could inform outcome measurement in practice. For example, where mental health care delivery and payment systems are structured around needs-based clinical groupings e.g. [72], different sets of core outcome measures could be considered for each group. Youth with mild or temporary difficulties who mainly require signposting or advice may benefit from the tracking of coping and self-management skills, in addition to symptom monitoring. In turn, tracking hopelessness, functioning, and suicidality may be indicated for youth with higher levels of distress and impairment, overlapping needs, or persistent risk.

### Study limitations

Several limitations must be noted. First, Q-methodology is not suited for informing generalisations about the distribution of viewpoints in the wider population [73]. It does, however, allow for generalisations “with respect to the subjectivity at issue”, that is about the existence of the identified profiles in one segment of the population [74]. This study does not claim that the outcome profiles identified are exhaustive or representative of all young people seeking help for depression in the UK, but provides a basis upon which new and more informed hypotheses can be built [32]. Notably, three Q-sorts were excluded from our interpretation of outcome profiles because they did not load significantly on any of the four principal components identified by the by-person factor analysis. This suggests that there may be additional outcome priority profiles that we were not able to characterise based on the size and composition of our study sample. All three participants whose Q-sorts were excluded belonged to ethnic minorities in the UK. This underscores the importance of giving special attention to minorities and hard-to-reach groups when examining outcome priorities in future studies, as these groups may have distinct outcome priorities.

We employed opportunistic snowball sampling [75] and participants self-selected into the study. Youth volunteers may have differed from the general population by having a particular interest in research. We aimed to mitigate this risk by recruiting from peer-support groups where individuals did not need to proactively contact the research team to arrange participation. Nevertheless, all study participants had successfully navigated their way towards some variation of mental health support and were willing to speak about this experience without fear of stigma. Youth to whom this does not apply may have different outcome priorities, which may have been missed.

As part of this study, a deliberate decision was made to prioritise young people’s self-report of lived experience, instead of formally screening for current depressive symptoms. The study’s broad inclusion criteria allowed us to include the views of young people who were not experiencing clinically significant depressive symptoms at the time of the research but felt that they could share relevant insights from previous treatment experiences. Self-reported information on the nature of young people’s lived experience with depression and other mental health difficulties was collected to contextualise outcome priorities, but this study was not designed to systematically assess associations between outcome priorities and clinical profiles. Future studies should examine such associations and investigate whether and how current symptom severity levels may influence young people’s outlook on priority outcomes.

This study was originally designed to include youth aged 12 to 21 years, but recruitment of younger adolescents proved difficult. They were rarely present in the peer support and youth advisory groups that we contacted although these tended to be open to this age group. Similarly, youth below the age of 16 did not respond to social media advertisements. The need to obtain parental consent was another logistical barrier to engaging adolescents under the age of 16 years [76]. Future studies may need to employ tailored recruitment strategies in order to gauge the views of this age group, for example by attempting to recruit these youth in clinical settings. Such research would be important to complement our study, as outcome priorities may vary by developmental stage. For example, younger adolescents might place a stronger emphasis on family relationships and parental support, compared with older adolescents.

## Conclusions

This study showcased considerable diversity in treatment outcome priorities among youth with lived experience of depression. Although all four outcome priority profiles prioritised symptomatic improvements such as improved mood and ability to feel pleasure, they differed with regards to the importance assigned to coping skills, the processing of past experiences and emotions, and functional impairment. These differences in outcome priorities appeared to be associated with varying degrees and types of complexity in youth’s mental health histories, as well as young people’s cognitive representations of depression and the perceived room of opportunity for improvement.

Outcomes other than symptomatic change are currently rarely measured in clinical research. This may lead to poor alignment between parameters used to judge treatment efficacy, and the needs and priorities of youth themselves. In clinical practice, ensuring that outcome measurement is acceptable, meaningful, and person-centered requires shared decision-making about the outcomes to track. While Core Outcome Sets can guide the measurement of essential consensus outcomes like symptom change, an additional element of personalisation is likely needed to reflect young people’s individual priorities.

## Supplementary Information

Below is the link to the electronic supplementary material.

Supplementary file1 (DOCX 71 kb)
