# Supplementary material for: What treatment outcomes matter most? A Q-study of outcome priority profiles among youth with lived experience of depression
Source: Eur Child Adolesc Psychiatry. 2021 Jul 17;32(1):123–37. doi: 10.1007/s00787-021-01839-x (PMC9908724; doi:10.1007/s00787-021-01839-x)
Supplement: Supplementary file 1 — Supplementary file1 (DOCX 71 kb) [file 787_2021_1839_MOESM1_ESM.docx]

**SUPPLEMENT**

What Treatment Outcomes Matter Most? A Q-study of Outcome Priority Profiles Among Youth with Lived Experience of Depression

K. R. Krause^1,2,3^, J. Edbrooke-Childs^1,2^, H. A. Bear^1,2,4^, A. Calderón^5^, M. Wolpert^2,6^

1) Evidence Based Practice Unit (EBPU), Anna Freud National Centre for Children and Families, The Kantor Centre of Excellence, 4-8 Rodney Street, London N1 9JH, UK

2) Research Department of Clinical, Educational and Health Psychology, University College London, Gower Street, London WC1E 6BT, UK

3) Cundill Centre for Child and Youth Depression, Centre for Addiction and Mental Health (CAMH), 80 Workman Way, Toronto, ON M6J 1H4, Canada

4) Department of Psychiatry, University of Oxford, Warneford Hospital, Warneford Lane, Oxford OX3 7JX, UK

5) School of Psychology, Universidad Gabriela Mistral, Avda. Ricardo Lyon 1177, Providencia, Santiago, Chile

6) Wellcome Trust, 215 Euston Rd, Bloomsbury, London NW1 2BE, UK

**Corresponding Author:** Karolin R. Krause, Cundill Centre for Child and Youth Depression, Centre for Addiction and Mental Health, 80 Workman Way, Toronto, ON M6J 1H4, Canada; Email: [karolin.krause@camh.ca](mailto:karolin.krause@camh.ca)

ORCID:

Karolin R. Krause: <https://orcid.org/0000-0003-3914-7272>

Julian Edbrooke-Childs: <https://orcid.org/0000-0003-0401-4058>

Holly A. Bear: <https://orcid.org//0000-0002-6737-6120>

Ana Calderón: <https://orcid.org//0000-0002-9984-6940>

Miranda Wolpert: <https://orcid.org/0000-0002-7463-4976>

**Table S1.** Demographic Characteristics and Self-Reported Mental Health History by Outcome Profile

| **Variable** | | **Profile A** | **Profile B** | **Profile C** | **Profile D** | **Not assigned** |
| --- | --- | --- | --- | --- | --- | --- |
| *N* |  | 6 (100.0) | 8 (100.0) | 8 (100.0) | 3 (100.0) | 3 (100.0) |
| Female | | 3 (50.0) | 3 (37.5) | 7 (87.5) | 2 (66.7) | 3 (100.0) |
|  |  |  |  |  |  |  |
| Mean age (SD) | | 18.2 (1.6) | 19.3 (2.2) | 18.8 (1.8) | 18.3 (2.3) | 19.3 (1.5) |
|  |  |  |  |  |  |  |
| Treatment history (based on self-report) | |  |  |  |  |  |
|  | Treatment ongoing (vs. ended) | 1 (16.7) | 4 (50.0) | 4 (50.0) | 2 (66.7) | 1 (33.3) |
|  | Repeated cycles of treatment (vs single cycle) | 3 (50.0) | 5 (62.5) | 4 (50.0) | 2 (66.7) | 1 (33.3) |
|  | History of admission to emergency care | 1 (16.7) | 0 (0.0) | 3 (37.5) | 2 (66.7) | 1 (33.3) |
|  | History of inpatient care | 0 (0.0) | 0 (0.0) | 1 (12.5) | 2 (66.7) | 3 (100.0) |
|  |  |  |  |  |  |  |
| Average number of additional difficulties (SD) | | 4 (1.79) | 2.6 (2.5) | 5.3 (1.8) | 7 (1.0) | 2.3 (2.1) |
|  | |  |  |  |  |  |
| History of additional mental health difficulties (based on self-report) | |  |  |  |  |  |
|  | Anxiety or phobia | 6 (100.0) | 5 (62.5) | 7 (87.5) | 3 (100.0) | 2 (66.7) |
|  | Sleeping problems | 4 (66.7) | 4 (50.0) | 5 (62.5) | 3 (100.0) | 2 (66.7) |
|  | Self-harm | 4 (66.7) | 3 (37.5) | 6 (75.0) | 3 (100.0) | 1 (33.3) |
|  | Eating problems | 4 (66.7) | 4 (50.0) | 4 (50.0) | 3 (100.0) | 1 (33.3) |
|  | Neurodevelopmental disorder | 1 (16.7) | 1 (12.5) | 4 (50.0) | 0 (0.0) | 1 (33.3) |
|  | Learning difficulties | 0 (0.0) | 1 (12.5) | 4 (50.0) | 1 (33.3) | 0 (0.0) |
|  | Anger and violent behavior | 2 (33.3) | 0 (0.0) | 2 (25.0) | 2 (66.7) | 0 (0.0) |
|  | Obsessions or compulsions | 1 (16.7) | 1 (12.5) | 3 (37.5) | 0 (0.0) | 0 (0.0) |
|  | Substance use | 1 (16.7) | 1 (12.5) | 2 (25.0) | 1 (33.3) | 0 (0.0) |
|  | Psychosis | 1 (16.7) | 1 (12.5) | 1 (12.5) | 2 (66.7) | 0 (0.0) |
|  | Trauma | 0 (0.0) | 0 (0.0) | 2 (25.0) | 2 (66.7) | 0 (0.0) |
|  |  |  |  |  |  |  |
| Types of treatment received (based on self-report) | |  |  |  |  |  |
|  | Individual psychotherapy or counseling | 6 (100.0) | 7 (87.5) | 6 (75.0) | 3 (100.0) | 2 (66.7) |
|  | Medication | 4 (66.7) | 3 (37.5) | 6 (75.0) | 2 (66.7) | 1 (33.3) |
|  | Family therapy | 2 (33.3) | 4 (50.0) | 4 (50.0) | 2 (66.7) | 1 (33.3) |
|  | Group therapy | 2 (33.3) | 2 (25.0) | 2 (25.0) | 2 (66.7) | 0 (0.0) |
